# Current Applications of Plant-Based Drug Delivery Nano Systems for Leishmaniasis Treatment

**DOI:** 10.3390/pharmaceutics14112339

**Published:** 2022-10-29

**Authors:** Darline B. dos Santos, Janaina A. Lemos, Sued E. M. Miranda, Leonardo D. Di Filippo, Jonatas L. Duarte, Lucas A. M. Ferreira, Andre L. B. Barros, Anna E. M. F. M. Oliveira

**Affiliations:** 1 Department of Biological and Health Sciences, Federal University of Amapá, Rodovia Juscelino Kubitisheck, km 02, Macapá 68902-280, AP, Brazil; 2 Department of Pharmaceutical Products, Federal University of Minas Gerais, Avenida Antonio Carlos, 6627, Belo Horizonte 31270-901, MG, Brazil; 3 Department of Drugs and Medicines, Sao Paulo State University, Rodovia Araraquara/Jaú, Km 01, Araraquara 14800-903, SP, Brazil; 4 Department of Clinical and Toxicological Analyses, Federal University of Minas Gerais, Avenida Antonio Carlos, 6627, Belo Horizonte 31270-901, MG, Brazil

**Keywords:** *Leishmania* control, neglected diseases, natural products, nanosystems

## Abstract

*Leishmania* is a trypanosomatid that causes leishmaniasis. It is transmitted to vertebrate hosts during the blood meal of phlebotomine sandflies. The clinical manifestations of the disease are associated with several factors, such as the Leishmania species, virulence and pathogenicity, the host–parasite relationship, and the host’s immune system. Although its causative agents have been known and studied for decades, there have been few advances in the chemotherapy of leishmaniasis. The urgency of more selective and less toxic alternatives for the treatment of leishmaniasis leads to research focused on the study of new pharmaceuticals, improvement of existing drugs, and new routes of drug administration. Natural resources of plant origin are promising sources of bioactive substances, and the use of ethnopharmacology and folk medicine leads to interest in studying new medications from phytocomplexes. However, the intrinsic low water solubility of plant derivatives is an obstacle to developing a therapeutic product. Nanotechnology could help overcome these obstacles by improving the availability of common substances in water. To contribute to this scenario, this article provides a review of nanocarriers developed for delivering plant-extracted compounds to treat clinical forms of leishmaniasis and critically analyzing them and pointing out the future perspectives for their application.

## 1. Introduction

*Leishmania* is a eukaryotic parasite from the order Kinetoplastida, belonging to the Trypanosomatidae family and responsible for causing leishmaniasis, one of the significantly neglected diseases worldwide [1]. It is a complex of diseases transmitted by species of mosquitoes of the genus Phlebotomus (New World) and Lutzomyia (Old World), resulting in health, social, and economic problems [2]. The genus Leishmania includes approximately 30 species, of which about 20 are zoonotic and pathogenic to humans. Among the species causing Leishmaniasis, we can highlight *L. donovani*, *L. chagasi*, *L. infantum*, *L. brasiliensis*, *L. amazonensis*, and *L. guyanensis*, among others [3,4]. The reservoir agents include mammals that comprise from humans to domestic (e.g., dogs) and wild animals (marsupials and rodents) distributed worldwide. This diversity of reservoirs and the high global prevalence become a barrier to controlling the disease [5].

The main drugs available for the treatment of leishmaniasis are pentavalent antimonials—meglumine antimony (Glucantime^®^) and sodium stibogluconate (Pentostam^®^). Other drugs that can be used are pentamidine and amphotericin B (AmB), but they have limited therapeutic results, often due to the microorganism resistance to the drugs. These drugs have problems relating to efficacy, cost, and significant toxicity in patients, as well as the long therapy regimes that make treatment a complex issue [6,7]. Analyzing the limitations faced by the current treatments for leishmaniasis, strategies to improve the effectiveness of the drugs should be used. One of these possible strategies would be the use of nanosystems for drug delivery [8].

Nanosystems are active drug delivery and pharmaceutical development at the nanometer scale (1–100 nanometers) that can be used for diagnosis, treatment, prevention, or theranostics [9,10]. They can protect drugs from degradation, alter pharmacokinetics and biodistribution, and decrease toxicity. Nanocarrier is a term used for pharmaceutical systems that can deliver hydrophilic and/or hydrophobic drugs, increasing their therapeutic efficacy and improving drug delivery to the target tissue [9,10,11]. These systems can be lipidic such as liposomes, nanoemulsions, nanostructured lipid systems, and nanostructured lipid carriers, among others. They can be polymeric such as micelles, nanoparticles, and dendrimers, or they can be metallic nanoparticles such as gold, zinc, and silver nanoparticles or carbon nanotubes, among other metals [12,13]. The choice of which nanoparticle to use depends on the drug used, form of administration, and expected biological advantages [13].

New strategies and recent advances to combat and control this important neglected disease are urgent. Thus, the demand for new treatments is necessary. The nanotechnology approach can help in the solubility of these compounds, mainly using natural compounds, decreasing the toxicity and improving uptake at the target sites. Considering this need for new therapeutic alternatives, in the present review, a special focus is given to nanosystem-loaded plant-based molecules for leishmaniasis treatment. An in-depth search of published studies was conducted using Web of Science, Science Direct, Springer, and Wiley, considering the names of nanosystems, *Leishmania* species, and natural products as keywords. Here, we briefly discuss the applications of these nanoparticles for in vitro or in vivo testing in leishmaniasis in the last 15 years.

## 2. Leishmaniasis—General Aspects

**Figure 1:**
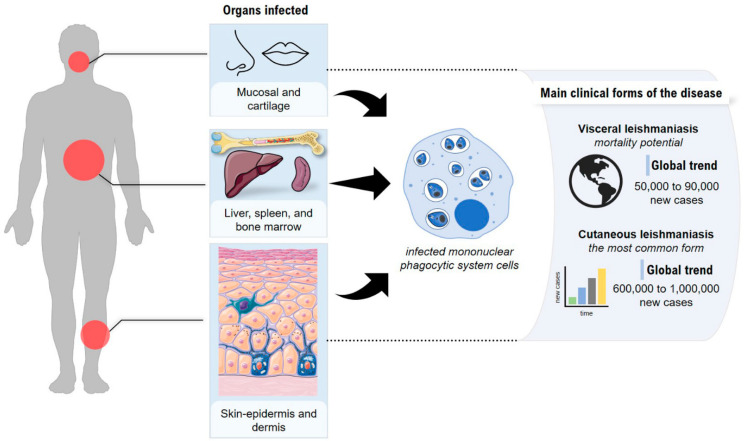
Forms of leishmaniasis and the tissues or organs affected. Visceral leishmaniasis: usually liver, spleen, and bone marrow become infected; cutaneous leishmaniasis: the skin becomes infected.

Leishmaniases are diseases with an elevated incidence rate and wide geographic distribution. They are endemic in about 98 countries located in tropical and subtropical areas, generally affecting low-income populations living in peripheries or rural areas. An estimated 350 million people live in risk areas for the disease, and 50,000 to 90,000 new cases of visceral leishmaniasis and between 600,000 and 1 million new cases of cutaneous leishmaniasis occur worldwide (Figure 1) [14,15]. It is believed that this estimate is not so realistic and that these numbers are much larger, mainly because of the large proportion of unreported or undiagnosed cases causing the disease to gain greater public attention due to its high rate of infection and morbidity [16].

Leishmaniasis is considered one of the six most important infectious diseases due to its high detection coefficient and the ability to produce deformities, especially in the tegumentary form, which leads to disfiguring lesions, triggering severe psychological, social, and economic problems [17]. In the visceral form of the disease, the involvement of internal organs results in complications that are often lethal [18].

**Figure 2:**
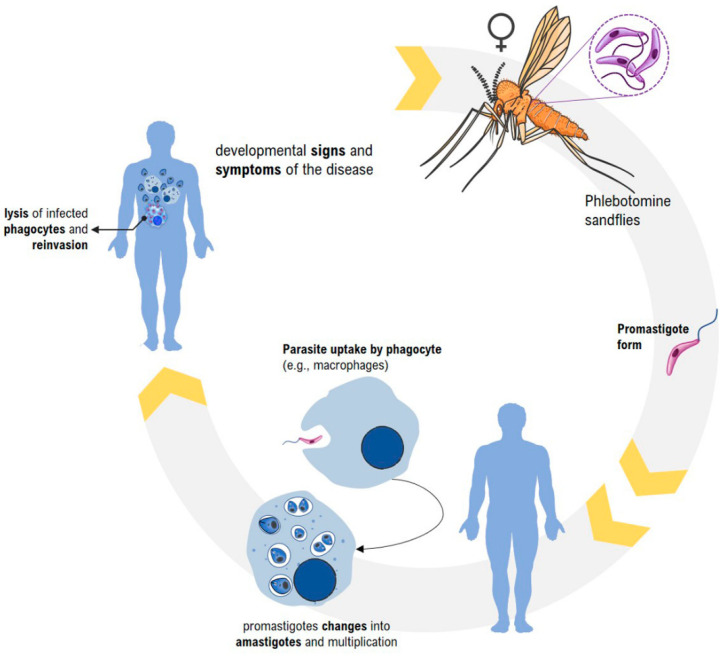
The stages of the Leishmania parasite in its life cycle.

Leishmania parasites are transmitted by the bite of infected phlebotomine sandflies. The parasite develops in the intestine of the female vector, which, when performing blood meal, transmits to the vertebrate host promastigotes metacyclic forms of the protozoan. After inoculation, the infectious forms are encompassed by cells of the host mononuclear phagocyte system, such as macrophages, which differentiate into amastigote forms, initiating a process of intracellular multiplication by binary division within the phagolysosome. When filled with the protozoan, the host cells rupture, releasing parasites that will infect new cells of the vertebrate [19,20]. When performing the hematophagy in an infected host, the mosquito ingests cells with the amastigote forms of the parasite. After the disruption of the morphonuclear and release of the protozoan forms in the organism of the vector, these become flagellated again, initiating a process of extracellular proliferation and therefore capable of infecting new reservoirs [21,22] (Figure 2).

Surveillance, prevention, and control actions require an excellent technical, operative, and political effort, given the high rates of morbidity and mortality related to the disease. Control measures are restricted to managing new and recurrent cases, entomological surveillance in zoonotic foci, and chemotherapy [23,24]. Thus, the advances in research to obtain vaccines and new drug therapies are of fundamental importance for the management and implementation of control measures. Vaccination could be an effective and essential strategy for long-term protection [25]. By limiting the severity and incidence, as well as interrupting the transmission, this prophylactic measure would reduce the cases of the disease [26].

For the treatment, parenteral administration of pentavalent antimonial is, for years, used against the clinical forms of the disease. However, several factors limit their use, such as drug resistance, high cost, toxicity associated with chemotherapy, adverse effects, and the inconvenience of the administration route (such as intravenous infusion). The use of AmB has also shown high rates of cure. However, its frequent side effects, such as nephrotoxicity and hypokalemia, besides the need for prolonged hospitalization for administration, limit its use. The effective and complete cure is of paramount importance since the disease, especially in its chronic form, is debilitating, making the patient stigmatized and incapacitated, and aggravating poverty, malnutrition, and other related disorders [6,27]. The urgency for more selective and less toxic drugs has led to several studies that have focused on the search for new drugs, improvement of existing drugs, and new routes of administration related to the treatment of leishmaniasis [28].

The chemical diversity of plant extracts makes them pharmacologically relevant for use as drugs. Many chemical isolates from natural products have already demonstrated considerable antiparasitic activity, evaluated through in vitro and in vivo studies, in the face of *Leishmania* species [29]. Secondary metabolites such as alkaloids [30], steroids [31], terpenoids [32], coumarins [33], and flavonoids [34,35], among others, revealed efficacy and selectivity against *Leishmania* species [36,37].

The study of new technologies within the health sciences provides several tools for research, such as therapeutic strategies that improve the administration of drugs and consequently their effectiveness. Faced with this scenario, much research has turned to the search for leishmaniasis drugs that may be less aggressive than the current reference treatments. Therefore, natural products in the form of oils, extracts, and fractions of specific compounds have been gaining emphasis [38,39]. Because natural products are not very soluble in water, the dispersibility of active compounds in aqueous media is reduced. In this context, the use of nanodelivery systems for natural products has stood out due to the advantages such as improvement in pharmacokinetics, solubility of lipophilic substances (such as vegetable oils), and protection of therapeutic substances against external agents [40,41]. In addition, nanostructured systems can be explored in various routes of administration, such as topically, for improving skin permeability [42,43], as well as oral or parenteral, for delivering the drug to target tissues [44]. Nanostructures such as metallic nanoparticles, lipidic nanoparticles, polymeric nanoparticles, liposomes, and nanoemulsions are the most used for the incorporation of vegetable oils [45,46,47].

## 3. Plant-Based Nanocarriers and Their Potential Application in Leishmaniasis Treatment

### 3.1. Nanoemulsions

Nanoemulsions (NEs) are systems consisting of immiscible liquids where one of them is dispersed in the other in the form of small droplets with particle sizes ranging from 20 to 500 nm [48,49]. This immiscibility creates a tension in the interface between the liquids, leading to thermodynamic instability of the system; for this, using surfactant and co-surfactants, even at low concentrations, it is possible to ensure kinetic stability, delaying the sedimentation or coalescence of the droplets [50].

NE has been an alternative to enable the use of insoluble or low-water-soluble substances, such as plant derivatives, presenting themselves as promising systems for the efficient delivery of drugs [51]. In addition, the reduced size of droplets allows for greater surface contact, facilitating better penetration of plant-based active ingredients, and confers several advantages to pharmaceutical formulations such as the improvement in pharmacokinetics, biodistribution of therapeutic agents, solubility of lipophilic substances, protection of active substances against the external medium, and possibility of use in different routes of administration [52,53].

The use of NE to improve the existing therapy of leishmaniasis has been reported. NE loaded with AmB showed a higher in vitro activity against life forms to *L. amazonensis*, besides showing fewer toxic effects against non-target cells when compared to the free form of the drug [54]. In another study, with BALB/c mice infected with *L. infantum chagasi*, the same AmB-NE was evaluated and showed promising results in the treatment of the visceral form of the disease. A decrease in the parasitic load in the liver and spleen was found, in addition to the absence of signs of toxicity when compared to the control group [44]. Other studies have shown the use of plant derivatives to obtain NE as leishmanicidal agents to optimize the treatment of clinical forms of leishmaniasis. These studies are summarized in Table 1.

As noted in Table 1, the development of plant-based NEs has been explored for studying leishmanicidal agents. In the Fabaceae family, one of the most representative of angiosperms, among genera that stand out as potential anti-leishmanial activity, is *Copaifera* spp., one of the most studied. Popularly known as Copaíba, it has been used in Brazil for centuries in folk medicine, especially in the Amazon region. Its oleoresin is rich in diterpenes and sesquiterpenes, providing the species with several biological properties such as anti-inflammatory [62] and antiprotozoal [63] effects. Some studies involving the use of free Copaiba oil indicate that components present in this plant derivative show promise for the treatment of leishmaniasis. This is case for diterpenes isolated from oleoresins of the genus that caused morphological and ultrastructural changes such as the appearance of rounded cells, disruption of the plasma membrane with loss of cell contents, and significant changes in the flagellar membrane in promastigotes of *L. amazonensis* [64]. In another study, ß-caryophyllene, a major sesquiterpene abundant in Copaíba, inhibited IFN-γ induced nitric oxide (NO) production in macrophages [65].

However, the intrinsic hydrophobicity of these compounds and the reported cytotoxicity of terpenoids could hinder their use in clinical therapy. The development of NE from copaiba might optimize the effect of these compounds. As shown in a study by Moraes and coworkers [58], in which the in vitro antileishmanial activity of Copaíba (*Copaifera* sp. Linnaeu) oil-loaded NE against promastigotes of *L. amazonensis* (strain MHOM/BR/M2269) and *L. infantum* (strain MHM/BR/1972/LD) was evaluated, the authors found IC_50_ of 30 ± 0.7 μg/mL and 18 ± 0.2 μg/mL, respectively, after 48 h of treatment. Infected macrophages treated by NE demonstrated a decrease in the infection rate of cells for both protozoan species. The in vivo study using infected BALB/c mice was conducted to determine whether NE can alter the course of infection in visceral and cutaneous leishmaniasis models. There was a reduction in the parasitic load and an improvement in the aspect of the lesions of the animals that received NE orally. In another study, a nanoemulsified carrier system prepared by mixing a surfactant, co-surfactant, Copaíba oil, and AmB was developed to obtain synergistic antileishmanial activity and improved oral bioavailability. The formulation was stable throughout the gastrointestinal tract and showed significantly improved in vitro antileishmanial activity, in addition to decreased hemotoxicity and renal toxicity compared to AmB [66].

Some studies against forms of *Leishmania* using the genus *Pterodon*, also belonging to the family Fabaceae, have shown positive results. Extracts from the fruits of *P. pubescens* were prepared using conventional methods and the supercritical fluid method. From them, NEs were developed and tested in vitro against promastigote and amastigote forms of *L. amazonensis*. The NE showed a better selectivity index and significantly improved activity against the parasites when compared to the non-encapsulated extracts. The authors suggested that the small size of the droplets allowed for better penetration of the active substances [56]. Studies indicate that the genus *Pterodon* has considerable anti-inflammatory activity, acting on the chemotaxis of mononuclear cells [67].

In another study, a *P. emarginatus* NE was produced with 20% of the oleoresin extracted from the fruits. Despite the high concentration of the oil resin present in the formulation, it presented a size and polydispersion index within the stability patterns. The use of this product topically in lesions induced by *L amazonensis* in BALB/c mice was able to improve histological parameters in the dermis and epidermis. The use of NEs also led to a decrease in the levels of the cytokines Il-10 and IFN-γ, as well as a reduction of the parasite load in the lesion, when compared to the control group, contributing to the end of the infectious process and the reduction of the size of the lesions [55,68].

To improve the penetration of the active compounds of *Citrus sinensis* essential oil into the skin, a NE was developed by a low energy intake method that was incorporated into a gel for transdermal topical application. The incorporation of NE into the gel, besides improving the viscosity of the product, allowed for a greater accumulation, favoring its penetration in the application site. The evaluation of the encapsulated product developed showed higher in vitro antileishmanial activity when compared to the free essential oil against the promastigote forms of *L. major* and *L. tropica* species that cause the cutaneous leishmaniasis in the Old World. The study suggested that small droplets of nanostructures may have better interaction with the membranes of the microorganism, favoring their destruction [59].

The NE developed with 10% clove oil (*Eugenia caryophyllus*) and sulfonamides dissolved in the oily phase acted effectively on promastigote forms of *L. infantum* and *L. amazonensis*. The NE could act by inhibiting a β-carbonic protozoan anhydrase. These metalloenzymes catalyze the reversible hydration of CO_2_ to bicarbonate with a proton release, suggesting a new mechanism of action in the destruction of microorganisms. In addition, it was observed that the product presented lower in vitro toxicity to RAW 264.7 macrophages and lower hemolytic activity when compared to AmB [69].

### 3.2. Liposomes

Liposomes (LPs) are spherical nanovesicles often composed of phospholipids and surfactants that spontaneously organize themselves into bilayers. In the bilayers, the individual lipid molecules are arranged in an orderly fashion with their hydrophobic tails facing inward and their polar groups facing toward the aqueous medium and the medium outside the liposome [70,71]. Because of the possibility of carrying both hydrophilic drugs (dispersed in the aqueous core or adsorbed on the surface) and lipophilic drugs (entrapped in the lipid bilayer), LP are considered drug delivery systems of wide application [72]. In addition, they are versatile structures because their characteristics such as size, lamellarity (number of bilayers), surface, fluidity, and composition can be modified according to the pharmacotechnical and pharmacological requirements necessary to transport the substance of interest [73].

Several reports in the literature suggest that LPs are also helpful in delivering plant-based derivatives for the treatment of leishmaniasis, as summarized in Table 2. As LPs are taken up by the mononuclear phagocytic system (MPS) predominantly by the macrophages in the liver and spleen, the main reservoirs of parasites in visceral leishmaniasis, their use has represented a promising strategy for the increasing concentration of drugs within these tissues, making the treatment against *Leishmania* infection more efficient [74].

Calvo and coworkers [75] recently developed LP obtained via the thin-film hydration method containing berberine (BER), an anti-inflammatory quaternary isoquinoline alkaloid extracted from *Berberis vulgaris* and *Berberis aristata*. BER exhibits antileishmanial activity in *L. donovani* promastigotes and amastigotes, triggering apoptosis-like cell death. In vitro cytotoxicity assays show that BER encapsulation reduces almost 10-fold the cytotoxic effect in healthy bone-marrow-derived macrophages. LP-BER also showed controlled release over time when compared to free BER. In vitro antileishmanial activity comparing free BER, LP-BER, and AmB (as positive control) showed a higher selectivity index for LP-BER, which means the LP formulation preferentially kills intracellular forms of *L. infantum* instead of the macrophages. Additionally, pharmacokinetic studies showed that LP-BER promotes higher blood circulation time and higher plasmatic concentrations than free BER in the BALB/c mice model. Furthermore, in a murine model of visceral leishmaniasis, LP-BER showed a significant reduction of parasites in the liver and spleen.

*Curcuma longa L.* has been historically related to microbial diseases due to its efficient antimicrobial properties, including against *Leishmania* [78]. In this way, Amaral and coworkers [76] also explored LP to enhance the therapeutic activity of curcuminoids against *L. amazonensis* promastigotes (Raimundo strain, MHOM/BR/76/Ma-5). The authors developed LP containing nonpolar hexane fractions of *C. longa L.* with or without cortex, separately. The major constituent of both fractions is ar-turmerone, which was considered a standard control for comparison effects. In vitro cytotoxicity against *L. amazonensis* promastigotes showed fractions with or without cortex had IC_50_ = 35.4 and 83 μg/mL, respectively, and ar-turmerone IC_50_ = 11 μg/mL. LP formulations containing the cortex or not were tested, and the IC_50_ values found were 0.4 and 2.9 μg/mL, respectively. It was possible to observe that the cortex fraction had superior anti-leishmanial activity. Due to volatile properties, ar-tumerone LPs were not successfully obtained. Morphological evaluation of untreated and LP-treated parasites showed LP formulation was able to promote serious morphological alterations possibly related to impaired vital functions and mitotic process, leading cells to death. Moreover, these data suggest LP encapsulation of curcuminoids can be a strategy to enhance their solubility and biological activity in the treatment of leishmaniasis.

Still studying curcuminoids’ liposomal formulations, Bafghi and coworkers [77] developed curcumin-loaded LP (LP-Cur) and tested the formulation in *L. major* (MRHO/IR/75/ER). LP-Cur was produced by thin-film hydration and showed controlled release over 3 days, releasing 40% of loaded curcumin. LP-Cur has not been shown to be toxic for HFF (human primary foreskin fibroblast cells). In vitro cytotoxicity against promastigotes showed that LP-Cur exhibits dose- and time-dependent toxicity; the highest dose tested (30 μg/mL of curcumin) killed 100% parasites after 24 h of treatment. The evidence suggests LP-Cur are a promising alternative to improve curcumin’s anti-leishmanial properties; a lower dose of curcumin could promote comparable in vitro cytotoxicity to currently prescribed drugs such as AmB.

Mandlik and coworkers [33] designed LPs to improve the water solubility and therapeutic activity of a compound screened from an in silico library. The compound 3-(1,3-benzodioxol-5-yl)-2-oxo-2H-chromen-6yl acetate (C2) showed the highest anti-leishmanial properties both in vitro and in vivo. The anti-leishmanial properties were assessed using *L. major* promastigotes. The IC_50_ ranged between 10 and 15 μM; at these concentrations, macrophages were 80–85% viable, indicating the LP-C2 formulation has no significant toxicity in healthy cells. After 48 h treatment, treated promastigotes had their morphology evaluated; the findings showed morphological alterations incompatible with life, causing notable alterations in size and motility. LP-C2 appears to significantly reduce mitochondrial membrane potential, indicating the cells are undergoing cell programmed death. LP formulation brought benefits observed in silico for the C2 compound and therefore can be further studied to treat cutaneous leishmaniasis.

Pentavalent antimonials have been the main drugs used to control leishmaniasis for more than half a century [79]. However, the systemic administration of antimonials has significant adverse effects on patients. To improve the treatment of cutaneous leishmaniasis caused by *L. amazonensis*, Lopes and coworkers [80] developed chloroaluminium-phthalocyanine-loaded LP for topical photodynamic therapy. LP was obtained by the thin-film hydration method followed by extrusion. *L. amazonensis* amastigotes (strain IFLA/BR/1967/PH8) were isolated from dorsal nodules of golden hamsters and inoculated in BALB/c female mice through subcutaneous injection at the base of the tail. After developing ulcerated lesions, animals were treated, covering wounds with 50 μL of formulations; after 15 min, the tissue was exposed to visible light irradiation at a wavelength of 660 nm, releasing 0–95 J/cm^2^ at an intensity of 81 mW/cm^2^ for 20 min. For comparison, a standard control group was treated with meglumine antimoniate (Glucantime^®^ at 200 mg Sb + 5/Kg/day) as well as the infected untreated animals. LP formulation was able to significantly reduce the parasite load compared to meglumine and untreated groups. LP formulation also promoted a significant reduction of parasite load in the spleen.

### 3.3. Lipid Nanoparticles

#### 3.3.1. Solid Lipid Nanoparticles

The first reports of solid lipid nanoparticles (SLNs) date from the early 1990s, as an alternative to conventional colloidal carriers—such as LP, polymeric microparticles, and microemulsion—for which the main goal was to improve oral bioavailability and the general biopharmaceutical properties of poorly water-soluble drugs. SLNs are solid-core nanoparticles extensively studied in the pharmaceutical field as drug delivery systems, obtained by a solid lipid dispersed in an aqueous phase within surfactants [81]. SLN has attracted major attention as novel colloidal drug carriers for application in leishmaniasis therapy. For instance, AmB-loaded SLN demonstrated efficacy against *L. major* infected BALB/c mice [82]. In another approach, plant-based nanoformulations of SLN showed refined performance against parasites of the *Leishmania* genus in vitro and in vivo (Table 3), highlighting the potential of SLN for the treatment of leishmaniasis [83,84].

Previous reports demonstrate that the lignoid fraction and the lipophilic compound yangambin (YAN), extracted from *Ocotea duckei* Vattimo, had promising in vitro activity against *L. amazonensis* and *L. chagasi* [85]. In this way, Marquele-Oliveira and coworkers [83] developed lignan fraction (LF)-loaded SLN from a methanolic extract of *Ocotea duckei*. SLN was prepared by the hot emulsification method. LF-SLN showed controlled release over 30 h. In vitro, healthy mammalian macrophages were used to assess the toxicity of LF-SLN, blank SLN, or free LF. For the highest concentration tested, free LF showed toxicity, while LF-SLN and blank SLN did not, indicating the encapsulation of LF from *Ocotea duckei* can reduce the toxic effects of free LF. In vitro anti-leishmanial activity using *L. amazonensis* promastigotes shows that within 24 h, LF-SLN released 45% of LF content and exhibited the same anti-leishmanial properties of free LF. Therefore, SLN seems to be an important tool, able to improve the use of LF, a Brazilian natural product, as an anti-leishmanial drug, reducing its toxicity in healthy macrophages and improving its cytotoxic properties against *L. amazonensis*.

#### 3.3.2. Nanostructured Lipid Carriers

Nanostructured lipid carriers (NLCs) are the second generation of lipid carriers that emerged around the 2000s as an alternative to overcome the limitations associated with SLNs [86]. This system can be obtained by replacing a fraction of solid lipid with oil (which is a liquid lipid at room temperature) that forms a complex lipidic matrix. The liquid lipid promotes a higher degree of matrix disorganization, allowing higher amounts of drug to be dissolved, as well as preventing drug leakage during storage, due to recrystallization phenomena as seen in SLN [87]. Table 3 shows plant derivatives loaded NLC for treating leishmaniasis.

**Table 3:** Plant-based solid lipid matrix (SLN and NLC) delivery systems as anti leishmanicidal agents.

Species	Vegetable Derivative	Physicochemical Characteristics	*Leishmania* spp.	Life Stage	IC50 (µg·mL^−1^)	In Vivo Effect	Toxicity	Reference
*Ocotea duckei Vattimo*	Yangambin lignan	Mean size: 218.8 nm, PDI 0.44, ZP: −30 mV, EE 94.2%	*L. amazonensis*	Promastigote	20	n.d.	Yangambin-loaded SLN reduces the toxic effects of free yangambin on macrophages (BMDM)	[83]
			*L. chagasi*					
Not showed	Ursolic acid	Mean size: 267 nm, PDI 0.18, ZP: −29.26 mV and EE 59%	*L. infantum*	Amastigote	n.d.	NLC showed the lowest parasitic load and superior activity to ursolic acid alone. No damage to kidneys and liver.	No histological or biochemical alterations were observed in healthy hamsters treated with the NLC	[84]
*Origanum vulgare*	Carvacrol	Mean size: 153 nm, PDI 0.2; ZP: between −20 and −30 mV	*L. amazonensis*	Promastigote	28.2	n.d.	IC_50_ for healthy THP1 cells was almost threefold higher than the IC_50_ for *L. amazonensis* promastigotes; the formulation was considered safe	[88]
				Amastigote	19.65			
*Cedar* threes	Cedrol	Mean size: 54.73 nm, PDI 0.254, ZP: −23.7 and EE 96.4 %	*L. donovani*	Promastigote	CRD-NLC improved cedrol anti-leishmanial activity—0.85- to 1.5-fold higher IC50 than free cedrol; CRD-NLC was twofold more selective to intracellular amastigotes	CRD-NLC reduced more than 99% of the parasitic load in the liver and spleen (two oral doses every 7 days). CRD-NLC had superior anti-leishmanial efficacy compared to free miltefosine at the same doses.	CRD-NLC were successfully internalized by macrophages without causing any damage	[89]
				Amastigote				
*Bixa orellana* L.	Seeds oil fraction	Size: 200 nm PDI 0.27 ZP: −30 mV spherical structures	*L. major*	Amastigote	Uptake of drug NLC, resulting in parasite elimination into the cell	n.d.	NLC loaded with 4% (*w*/*w*) seed oil showed IC_50_ of 153.6 and 123.4 mg/mL for 3T3 and HaCaT, respectively. Efficacy against *L. major* (concentration 2 and 5 µg/mL) was considerably lower than the concentrations that caused toxicity to 3T3 and HaCaT.	[90]

Abbreviatures: polydispersity index (PDI); zeta potential (ZP); encapsulation efficiency (EE); fibroblast’s cell (3T3); keratinocyte cell (HaCaT); bone-marrow-derived macrophages (BMDM); human monocytic cell line (THP-1); not determined (n.d.); solid lipid nanoparticle (NLS); nanostructured lipid carrier (NLC); cedrol (CRD).

Recently, a study reported the potential of NLCs loaded with annatto oil fraction (AO) from *Bixa orellana* L. for the treatment of cutaneous leishmaniasis [90]. The NLCs showed improved stability, without significant changes in physicochemical parameters, over the period studied (90 days). As protozoa of the genus *Leishmania* are obligate intracellular parasites with tropism for the polymorphonuclear cells of mammals, the treatment of the disease must be designed to target these internalized life forms [91]. Thus, this study evaluated the effects of AO and AO-NLCs on the parasitic load of macrophages, wherein the use of AO-NLCs showed better anti-amastigote effects than AO, probably due to enhancing cell permeation and internalization of the AO in macrophages previously infected with *L. major*. Furthermore, possible toxic effects of AO-NLCs were evaluated against fibroblasts and keratinocytes, and the results indicated a large margin of safety for the proposed formulations [90]. Despite promising results, further studies should be carried out to elucidate possible mechanisms of AO-NLCs involved in cytotoxicity in macrophages and other cell types.

**Figure 3:**
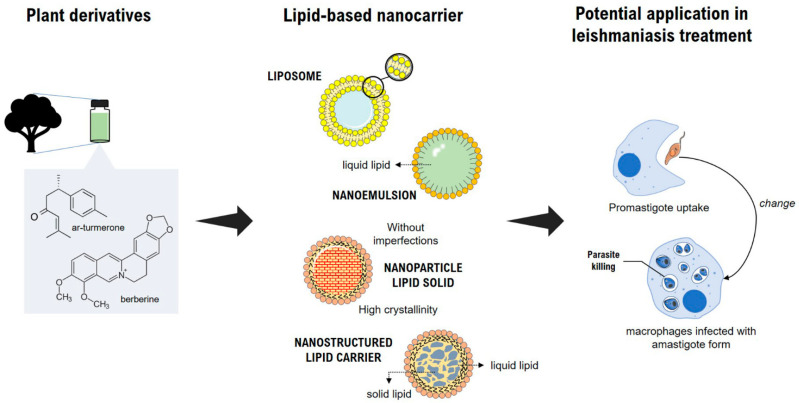
Schematic illustration of plant-based lipid nanocarriers used as potential anti-leishmania activity.

Enhanced antileishmanial activity of plant-based lipid colloidal drug carriers such as NE, LP, SLN, and NLC are emerging as a promising approach because the nanocarriers are readily internalized by macrophages in the liver and spleen (Figure 3), releasing the drug inside the cell and thus leading to a high local concentration, ultimately killing the parasites [92,93,94,95]. In this regard, the main strategy in treating *Leishmania* is to enhance macrophages’ drug targeting, using enhanced nanosized delivery systems. Another usual approach to also increase parasite selectivity is active targeting, which involves modification of the nanocarrier surface through a ligand for the receptor expressed by macrophages [87,96].

### 3.4. Polymeric Nanoparticles

Polymeric nanoparticles (NPs) are nanosized colloidal particles that possess properties such as a large surface-to-volume ratio, small size, and tunable surface chemistry that provide them with advantages over their bulk materials [97]. The term nanoparticle includes nanocapsules and nanospheres. Nanocapsules consist of a polymeric shell disposed around an oily core, and the drug may be dissolved in this core and/or adsorbed to the polymeric wall. On the other hand, nanospheres are formed by a polymeric matrix, wherein the drug can be physically and uniformly retained or adsorbed [98]. Thus, a diverse group of NPs can be developed predicated upon the rational design of polymers tailored for specific drug load, facilitating their delivery to the site of action and being engineered to meet distinct biomedical applications [99].

**Figure 4:**
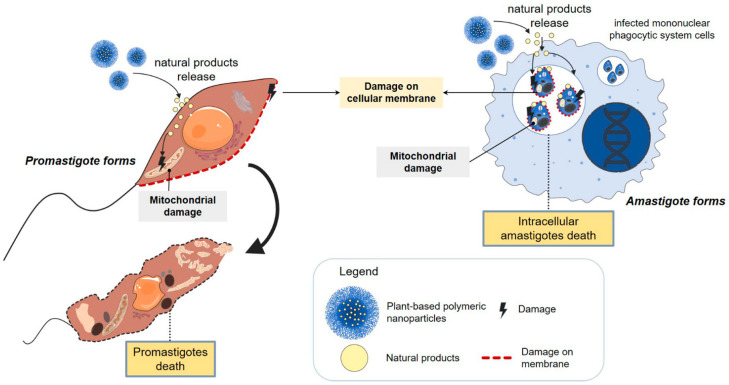
Schematic representation of the association of plant-based natural products with polymeric nanoparticles. Polymeric nanoparticles loaded with plant-based products allow for targeted and efficient delivery of anti-Leishmania agents, promoting changes in cellular structures and mitochondrial damage, leading to the death of promastigote and the intracellular amastigote forms.

During the last decade, polymer-based drug delivery systems have been used to enhance the performance of drugs in the treatment of leishmania [100]. In the same sense, the combined use of NPs with anti-leishmania natural products has been the target of research for the development of new treatments, as these nanocarriers can penetrate the macrophage cells and reach the infectious parasite, enabling targeted and efficient delivery [101]. Furthermore, natural compounds have shown the ability to generate changes in cellular structures, including at the mitochondrial level, leading to the death of promastigote and the intracellular amastigote forms [102,103,104,105] (Figure 4).

The studies using polymeric nanoparticles in the treatment of leishmaniasis are summarized in Table 4.

PLGA nanoparticles carrying the saponin beta-aescin were prepared by a W/O/W (water/oil/water) emulsification solvent evaporation. These NPs presented higher cytotoxicity against MRC-5 cells and macrophage-like J774 cells when compared to the blank NPs, showing a higher selectivity index (18, compared to 4 in blank NP) and presenting potential as a drug delivery platform for antileishmanial drugs [106].

In another study, NPs were made of poly-ε-caprolactone (PCL) and pluronic for the delivery of red propolis ethanolic extract (EPE). The NPs presented similar IC_50_ to the EPE (31.2–47.2 μg/mL and 38.3 μg/mL, respectively) against *L. brasiliensis* promastigotes. The main chemical composition of EPE was flavonids formononetin, isoliquiritigenin, and liquiritigenin [110]. Nascimento et al. (2016) [104] showed similar results in PCL-red propolis nanoparticles. The propolis extract presented an IC_50_ of 37.9 μg/mL, and the nanoparticles with the best results showed an IC_50_ of 31.34 μg/mL. Flavonoids are known for presenting leishmanicidal activity acting by Arginase inhibition [105,111]. Moreover, flavonoids can interfere with iron metabolism, targeting ribonucleotide reductase [39].

For the delivery of betulinic acid (BA), pentacyclic triterpenoid chitosan nanoparticles were developed by Mehrizi et al. (2018) [107] with a sustained and slow release of BA; additionally, the NPs were successfully internalized by macrophages. In vitro studies indicated that NPs decreased the *L. major* amastigotes and promastigotes viability. In vivo toxicity studies showed that BA encapsulation led to less toxicity, and 20 mg·kg^−1^ doses were selected for efficacy studies. In these studies, the results show that BA NPs are effective in reducing parasite load and lesion size. In another study, chitosan nanoparticles were developed for *Matricaria chamomilla* essential oil loading (CEO) [108]. In vitro, biological studies showed that the CEO NPs maintained the activity against the promastigote and amastigote forms of *L. amazonensis*. Additionally, CEO NPs had a high selectivity index (SI > 10) for macrophages infected with intracellular parasites forms; this can be explained by the macrophage’s ability to uptake the chitosan nanoparticles as observed in other studies [103,112]. On the other hand, the CEO NPs also showed lower cytotoxicity than free essential oil against mammalian cells, suggesting a great potential for anti-leishmanial therapy [108].

*Nigella sativa* oil PCL nanoparticles against *L. infantum* promastigotes and amastigotes in vitro were developed by Abamor et al. in 2018 [109]. *N. sativa* oil NPs initially exhibited slow release, increasing considerably over time after 72 h. The NPs presented no cytotoxicity on J774 macrophage cells. On the other hand, the free oil presented a reduction in J774 macrophage cell viability. The *N. sativa* oil NPs inhibited more than 80% of *L. infantum* promastigotes and amastigotes after 192 h incubation, dramatically suppressing the infection indexes of macrophages.

These promising data reveal that plant-based nanoparticulate delivery systems can be successfully used as a strategy to fight leishmaniasis infection soon after the determination of their in vivo activities.

### 3.5. Metallic Nanoparticles

Lately, inorganic compounds as delivery systems are receiving considerable attention in the pharmaceutical field. Metallic nanoparticles (MNP) are nanosized particles made of metals such as zinc, iron, gold, and silver that offer a wide range of applications in therapeutics, biotechnology, gene delivery vehicles, and drugs. Due to their magnetic and mechanical properties, as well as specific technological characteristics such as melting point and surface area, MNPs have become a promising approach for better diagnostic devices and nanomedicines [113].

Green synthesis has attracted attention for the synthesis of various metal and metal oxide nanoparticles as a reliable, sustainable, and eco-friendly option. Among the available green methods of synthesis for metal/metal oxide nanoparticles, utilization of plant extracts is a rather simple and easy process to produce nanoparticles biogenic at a large scale relative to bacteria- and/or fungi-mediated synthesis. Plants have biomolecules (such as carbohydrates, proteins, and coenzyme) with exemplary potential to reduce metal salt into nanoparticles [91]. Studies have shown that metal oxide nanoparticles have great potency in the treatment of leishmaniasis (Table 5). Green nanotechnology provides excellent opportunities to produce MNP with high stability, toxic-free abilities, and antioxidant properties [114]. In different studies, it was shown that green nanoparticles displayed high anti-leishmanial activities on both promastigote and amastigote forms of *Leishmania* parasites.

Gold nanoparticles (AuNPs) using the aqueous extract of *Rhazya stricta decne* as a source of reducing and stabilizing agents were produced by Ahmad et al. in 2017 [115]. The nanoparticles presented no significant toxicity in THP-1 macrophages when compared with the control cells’ viability (97.42% ± 1.4). When tested against intracellular amastigotes of *L. tropica* clinical isolate ANWARI, the microparticles reduced the number of amastigotes with an increasing concentration of AuNPs. Gold and silver nanoparticles were biosynthesized using *Olax nana* Wall. ex Benth aqueous extract and presented lightly hemolytic activity (5–45 μg·mL^−1^), as well as non-toxic against freshly isolated human macrophages, showing their compatibility with the biological environment. Exposure of leishmanial cells to silver–gold nanoparticles produced a concentration-dependent inhibition, and the lower IC_50_ values indicated their potential for treatment of promastigote and amastigote stages of *L. tropica* (KMH23) [116]. In a similar study, gold–silver nanoparticles presented concentration-dependent cytotoxicity in *L. donovani* promastigotes, leading to a reduction of 45% versus 62% when using miltefosine [117]. The significant antileishmanial results of gold samples may be attributed to a possible inhibitory effect on some of the vital enzymes of amastigotes, induction of apoptosis, and generation of intracellular reactive oxygen species (ROS) without inducing death in the macrophage population [110,117].

*Rhamnus virgata* extracts have also been employed successfully to prepare cobalt and chromium oxide nanoparticles with enhanced antileishmanial properties, besides being considered nontoxic to macrophages and being hemocompatible [118,119]. Due to their enhanced volume-to-surface area ratio, improved optical properties, and capacity for ROS production, zinc oxide nanoparticles (ZnONPs) have been studied in relation to their antileishmanial effects. In a study conducted against *L. tropica* promastigotes, microparticles biosynthesized from plants from the genus *Verbena* presented promastigote inhibition in a concentration-dependent way, with 60% of mortality in the highest concentration (250 µg/mL) [120]. Like these finding, ZnONPs synthesized from *Silybum marianum* presented antileishmanial activity against promastigote cultures of *L. tropica* KMH23 (Khyber Medical Hospital-23), presenting an 80% promastigotes inhibition at a concentration of 1000 µg/mL [121].

Silver nanoparticles (AgNPs) also are applied against leishmaniasis. AgNPs synthesized with *Mentha longifolia* (L.) aqueous extract presented an IC_50_ of 8.73 µg/mL against *L. tropica* promastigote. The nanoparticles lead to the formation of ROS, which can be related to the anti-leishmanial potential of the AgNPs [122]. In another study, AgNPs synthesized with *Euphorbia prostrata* extract were tested against promastigotes and amastigotes of *L. donovani* (strain MHOM/IN/80/DD8). The AgNPs presented an IC_50_ of 4.94 µg/mL in promastigotes and 3.89 µg/mL in intracellular amastigotes as well as pronounced morphological changes in the parasites. A decreased ROS level was also observed, which could be responsible for the observed caspase-independent shift from apoptosis to massive necrosis, which might be considered a possible mechanism of action for AgNPs [123]. Similarly, AgNPs synthesized from ginger rhizome were able to promote apoptosis in promastigote parasites from *L. major* (60.18%) and indicated an acceptable level of in vitro activity against intracellular amastigotes [124]. However, the research suggests that further efforts should be made to identify the mechanisms of antileishmanial activities of these nanoparticles in vivo.

In vivo, the AgNPs showed potential in the treatment of a murine model of *L. major* infection. The AgNPs were obtained using *Moringa oleifera* aqueous extract. The oral treatment of mice infected with *L. major* resulted in a significant reduction in the average size of leishmaniasis cutaneous lesions compared with untreated mice. The treatment led to a reduction in lipid peroxidation, NO, and increased glutathione levels, indicating an antioxidant property of AgNPs [125].

In the search for strategies to improve the treatment of leishmaniasis, combination regimen therapy has been a promising outcome. In this regard, the combination of miltefosine with AgNPs synthesized from *Anethum graveolens* aqueous extract magnifies the antileishmanial effect of miltefosine by about twofold against promastigote parasite stage [126]. This enhancive effect of AgNPs on miltefosine activity may be attributed to an apparent loss of structural integrity and DNA fragmentation, inducing apoptosis-like death. Furthermore, the increased antileishmanial activity in intracellular amastigotes could be mediated by the spherical shape of the AgNPs or even the variation in surface total charge that might facilitate AgNPs’ entry into the cells. Similarly, the combination of silver-doped titanium dioxide nanoparticles and *Nigella sativa* oil enhanced the inhibitory effects of promastigotes and amastigotes stages of *L. tropica* while being shown to be non-toxic against macrophages cells, in contrast to the application of isolated substances [102]. As it is seen, combination therapy can be an effective and safe opportunity to treat leishmaniasis, as well as the use of nanotechnology-based formulations, which have shown promising results.

**Table 5:** Plant-based metallic nanoparticles as antileishimanicidal agents.

Species	Vegetable Drivative	Physicochemical Characteristics	*Leishmania* spp.	Life Stage	IC50 (µg·mL ^−1^)	In Vivo Effect	Toxicity	Reference
*Nigella sativa*	Essential oil	TiAgNps—n.d.	*L. tropica*	Amastigote	n.d.	n.d.	no cytotoxic for J774A.1 macrophages	[102]
				Promastigote				
*Rhazya stricta decne*	Aqueous extract	AuNPs—size: 40 nm ZP: −46 mV	*L. tropica*	Amastigote	43	n.d.	biocompatible with THP-1 macrophages	[115]
*Olax nana* Wall. ex Benth	Leaves -aqueous extract	AgNPs— size: 31 nm ZP: +28 mV PDI 0.31 spherically shaped	*L. tropica*	Amastigote	17.44	n.d.	shown to be non-toxic to macrophages and compatible with the biological environment	[116]
				Promastigote	12.56			
		AuNPs Size: 65 nm ZP: +32 mV PDI 0.51 spherically shaped		Amastigote	42.20			
				Promastigote	21.52			
*Trigonella foenum-graecum*, *Coriandrum sativum*, and soybean	Leaf extracts	Au-Ag BNPs—size: 10–12 nm, monodispersed, spherically shaped	*L. donovani*	Amastigote	46% reduction, 31% reduction, and 45% reduction in BNPs of macrophages treated with soybean, fenugreek, and coriander leaf extracts, respectively	n.d.	biocompatible with THP-1 macrophages	[117]
				Promastigote	0.04			
*Rhamnus virgata*	Leaf extract	CoONPs—Size: 123.9 nm ZP: +26.9 mV PDI 0.215	*L. tropica*	Amastigote	58.63	n.d.	compatibility with the biological environment	[118]
				Promastigote	32.64			
*Rhamnus virgata*	Leaf extract	Cr_2_O_3_NPs—size: 274.1 and 1.91 nmZP: +45.5 mV PDI 0.505	*L. tropica*	Amastigote	44.31	n.d.	nontoxic macrophages and biocompatible	[119]
				Promastigote	33.24			
				Promastigote	24.9			
*Verbena officinalis*	Leaf extracts	ZnONPs—size: 14–31 nm rod and flower shapes	*L. tropica*	Promastigote	243	n.d.	n.d.	[120]
*Verbena tenuisecta*		Size: 65–75 nm rod and flower shapes			414			
*Silybum marianum*	Aqueous extract	ZnONPs—size: 25 nm ZP: −2.28 mV spherically shaped	*L. tropica*	Promastigote	246	n.d.	n.d.	[121]
*Mentha longifolia* (L.) L.	Leaves—aqueous extract	AgNPs—size: <100 nm PDI: 0.263 spherically shaped	*L. tropica*	Promastigote	8.73	n.d.	n.d.	[122]
*Euphorbia prostrata*	Leaves—aqueous extract	AgNPs—size: 12.82 nm polydisperse spherical and irregular shaped	*L. donovani*	Amastigote	14.94	n.d.	no cytotoxic for J774A.1 macrophages	[123]
				Promastigote	3.89			
*Zingiber officinale*	Rizhome—aqueous extract	AgNPs— size 10 nm approximately spherical	*L. major*	Amastigote	2.35 (ppm)	n.d.	high concentrations have more toxic impacts on macrophage RAW.264.7 (≥ 2.5 ppm)	[124]
				Promastigote	n.d.			
*Moringa oleifera*	Leaves—aqueous extract	AgNPs—size: 116.2 nm PDI: 0.2 spherical shaped	*L. major*	Promastigote	n.d.	lesion size and complete healing after 14 days	minimized ROS-mediated toxic effect	[125]
*Anethum graveolens*	Leaves—aqueous extract	AgNPs—size: 35 nm spherical shaped	*L. donovani*	Amastigote	n.d.	n.d.	biocompatible with RAW 264.7 macrophages	[126]
				Promastigote				
*Maytenus royleanus*	Stem—aqueous extract	AuNPs—mean size of 30 nm, ZP of −42.9 mV, and hexagonal shape	*L. tropica*	Promastigote	AuNPs inhibited the parasite growth by 75% after 72 h of incubation	n.d.	n.d.	[127]
				Promastigote				
*Elaeagnus angustifolia*	Leaf extracts	ZnONPs—size: 205.9 nm, a ZP: 13.8 mV and a PDI 0.132	*L. tropica*	Amastigote	32.83	n.d.	n.d.	[128]
				Promastigote	24.9			
*Callistemon viminalis*	Floral extracts	NiONPs—size: 20 nm–22 nm (300 °C); 16 nm–17 nm (400 °C), and 14–16 nm (500 °C)	*L. tropica*	Promastigote	37	n.d.	n.d.	[129]

Abbreviatures: polydispersity index (PDI); zeta potential (ZP); silver nanoparticles (AgNPs); gold nanoparticles (AuNPs); cobalt oxide nanoparticles (CoONPs); gold–silver bimetallic nanoparticles (Au-Ag BNPs); chromium oxide nanoparticles (Cr2O3NPs); zinc oxide nanoparticles (ZnONPs); nickel oxide nanoparticles (NiONPs); silver-doped titanium dioxide nanoparticles (TiAgNps); not determined (n.d.).

## 4. Possible Mechanisms Involved with Increased Antileishmanial Activity by Nanosystems

**Figure 5:**
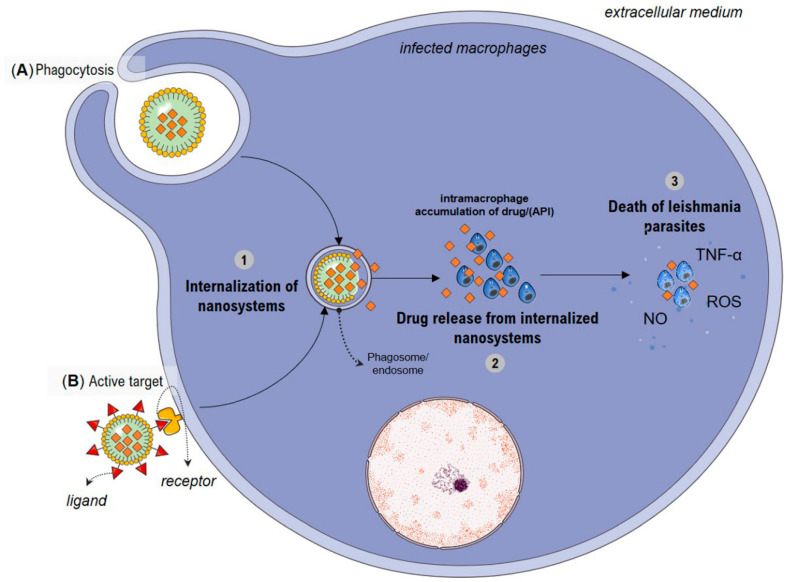
General illustration of possible mechanisms involved with nanosystem-based antileishmanial activity. The enhanced internalization of nanosystems into Leishmania-infected mononuclear phagocytic system cells mediated by (**A**) phagocytosis or (**B**) active target with the aim of achieving selective accumulation inside the cells with release of the active pharmaceutical ingredient (API) promoting a leishmanicidal effect on parasites. Abbreviations: API (active pharmaceutical ingredient); NO (nitric oxide); ROS (reactive oxygen species); TNF-α cytokine (tumor necrosis factor alpha).

The increased efficacy in the treatment of leishmaniasis in vitro and in vivo observed in previous studies may be based upon the enhanced cellular internalization and trafficking of designed nanosystems. The enhanced internalization into macrophages could result in sufficient accumulation to eradicate the *Leishmania* parasites [8]. Indeed, infected macrophages are expected to recognize active pharmaceutical ingredient (API)-loaded nanoparticles, which undergo phagocytosis (Figure 5A), releasing the API inside the macrophages and promoting the leishmanicidal effect directly in the parasites [94]. Usually, the enhanced cellular internalization and trafficking are attained through alteration in physicochemical, in other words, modifying its size and shape (morphology) or surface structure [92].

Several nanosystems with particle sizes ranging from 50 to 200 nm are associated with increased phagocytosis and can be internalized by macrophages infected with *Leishmania* [93,130,131]. The NP shape is a factor that also appears to have a pivotal role in their internalization. Xie and coworkers [132] observed that triangular NPs undergo significant accumulation in RAW264.7 cells as compared to other shapes (stars, rods). On the other hand, spherical gold NPs had better uptake by macrophages than rod-shaped NPs [133,134]. Concerning surface charge, nonphagocytic cells adsorb cationic nanoparticles to a higher extent, while phagocytic cells preferentially take up anionic nanoparticles [135]. Thus far, the cellular uptake of nanoparticles by macrophages and their further interaction with the immune system appears to be markedly dependent on size, shape, and surface charge. Another property is hydrophobicity, which influences the control of the absorption and distribution of nanocarriers, cell interactions, protein interaction, and particle clearance [101].

In studies conducted with nanosystems associated with natural products of plant species, some mechanisms of action were hypothesized. In a study by Moraes et al. in 2018 [58], ultrastructural analyses by scanning electron microscopy showed that andiroba and copaíba NE acted on the membrane of the promastigote forms of *L. amazonensis* and *L. infantum*, promoting a change in parasite morphology and the retraction of the scourge. This process would alter the mobility of the parasitic form by decreasing its capacity for infection.

However, to ensure successful treatment of leishmaniasis, strategies such as active targeting can be opted for the purpose to achieve selective drug accumulation inside the organs, tissues, or cells of interest (Figure 5B). In this sense, promising results from lipid nanoparticles covered with chitosan were presented [130]. Chitosan attachment exteriorly to the NLC enhances its recognition for having a unit of D-glucosamine, which targets galactosamine/glucosamine-like receptors in the membrane of tissue macrophages. All these events lead to a significant uptick in NP intramacrophage accumulation; however, for plant-based nanosystems used in the treatment of leishmaniasis, active targeting has not been developed. This fact may be due to each component of a complex nanosystem participating in many different interactions, and these interactions generate unforeseeable, emergent properties not properly understood [136].

It is known that the leishmanicidal activity of the nanosystems depends on the rate of cellular uptake by macrophage cells. Therefore, API release is considered a critical step in the elimination of intracellular *Leishmania*. The mechanism that will cause the death of these parasites will depend on each encapsulated active compound [137]. Studies showed that the antileishmanial activity of cedrol-loaded NLC, a triterpene, is based on their ability to modulate NO production precisely targeting the macrophage phagosomal *Leishmania* parasite [89]. It has been described that NO and ROS are essential molecules produced by macrophages for incapacitating and destroying intracellular pathogens [138]. Recently, Assolini and coworkers [139] also noted that the chalcones loaded in NLS induced ROS, NO, and cytokines (TNF-α) production, corroborating with microbicidal potential and leading to the reduction of intracellular amastigotes. Although studies show that NO and ROS are the main mediators for killing *Leishmania* in mice, possible mechanisms of action of plant-based nanosystems should be better investigated on amastigote parasites [117,140] in order to provide an additional impetus to the ongoing search for novel drugs for effectively managing of leishmaniasis.

Despite parenteral administration of pentavalent antimonials being the treatment used for all forms of leishmaniasis, it has several limitations. Drug delivery systems have been increasingly investigated as a promising alternative to treatments. Several mechanisms may be related to the more effective delivery of drugs and the death of protozoa. Leishmaniasis has different clinical manifestations that could allow for treatment by other routes of administration. For example, in the case of tegumentary leishmaniasis, where in general, the initial phase of the disease is in the dermis layers, the use of nanosystems, due to the small size of their particles, may favor the penetration of lipophilic compounds, as well as improve and increase dermal permeability and bioavailability, increasing their topical effect [141]. In this case, topical treatment is an attractive alternative to the cutaneous forms of the disease and other pathways such as orally to other forms of the disease, offering significant advantages over intravenous therapy: fewer adverse effects, ease of administration, and lower costs. The use of nanotechnology associated with the treatment of leishmaniasis could allow bioactive molecules by different routes.

## 5. Perspectives and Conclusions

Currently, there is great interest in the study of natural products in the development of nanosystems in the therapy of leishmaniasis, especially plant extracts and oils. The evaluation of the activity of plant derivatives is complex and should consider several factors, such as the type of extraction, the polarity of the solvent used, and which part of the plant was used—because one can find different chemical compositions [142]. Thus, studies of characterization and standardization of plant extracts are critical for understanding the action of the specific groups presented in this review. The antileishmanial activity of analogs and compounds isolated from plants is promising and becomes an interesting strategy for the discovery of new leishmanicidal compounds [143].

In addition to information related to plant products, it is essential to consider the parasite species (or the strain) in evaluating antileishmanial activity. In most studies, only the in vitro activity of nanosytems against Leishmania stages is evaluated, and this is most often not sufficient for the development of effective new drugs. There are few in vivo studies related to leishmaniasis and parasitic cycle using plant derivatives. They would be important because they evaluated several factors besides the parasite’s susceptibility, such as the drug’s ability to be internalized by phagocytic cells, the resistance of the molecule to intracellular degradation, and the possibility of cytotoxicity. Even in vitro studies, are of paramount importance in evaluating disease-related activity, its mechanisms of action, and death, taking into account the shape of the tested parasite and also investigating possible toxicity to vertebrate host cells. Another point to be addressed is that, despite the growing interest in the search for promising treatments using these nanosystems, no clinical study has yet been reported.

In addition, the compound’s activity against the protozoan can be different depending on the stage of the parasite. It is known that the life cycle of *Leishmania* is dimorphic. It has the extracellular flagellated promastigote form that multiplies and survives exclusively in the insect. The amastigote form is a mandatory intracellular form of polymorphonuclear cells of mammals. Two forms of the same agent require different environmental and physiological conditions and, consequently, react specifically against each leishmanicidal agent and may respond differently to each compound or plant species evaluated [144]. Studies using amastigote forms reflect the situation of the disease in the mammalian organism, and this should be taken into account since it is the stage of the most relevant parasite. The activity in the promastigote form, on the other hand, provides essential information on the specificity of the tested agent, serving as a preliminary screening to identify new compounds. However, this is not a form that manifests itself in mammals, from the clinical point of view of the disease [145].

The high morbidity and mortality rates of leishmaniasis combined with the fact of limitations of use and toxicity of available drugs encourage the search for new therapies that are safer, more efficient, and less expensive. Natural resources of plant origin are potential sources of new molecules, thus arousing the interest of researchers. The screening and obtaining of new, active plant-based biomolecules draw much attention since they have complex chemical composition, as well as lower toxicity and risk of resistance to microorganisms, being, therefore, a target for the production of new drugs, including antileishmanial agents. To determine the efficacy of a compound against leishmaniasis, in vivo tests are required, as well as the evaluation of the best mode of administration of the compound is important.

Therefore, the innovations proposed in view of the complexity of natural compounds inserted in nanostructured formulations with a sustained release system and reduced cytotoxic capacity for positive effects against leishmaniasis, whether additive or synergistic inhibitory, follow important scientific paths for investigation and alternatives for treatment.

## Figures and Tables

**Table 1:** Relationship of plant-based nanoemulsions as leishmanicidal agents.

Species	Vegetable Derivative	Physicochemical Characteristics	*Leishmania* spp.	Life Stage	IC50 (µg·mL^−1^)	In Vivo Effect	Toxicity	Reference
*Pterodon emarginatus* vogel	Fruits—oleoresin	Mean size: 158.10 nm, PDI 0.136	*L. amazonensis*	Amastigote	n.d.	Decrease in lesion diameter; improvement in histopathological parameters, reduction in parasite load, and reduction in cytokine levels.	No cytotoxic effect in vitro	[55]
*Pterodon pubescens* benth	Fruits—extracts	Mean size: 185 nm, PDI 0.170, and consistency index of 1405.00	*L. amazonensis*	Amastigote	1.9 ± 0.30	n.d.	Moderate cytotoxicity against J774	[56]
*Copaifera paupera*	Trunks—oleoresin	Mean size: 114.9 ± 1.2 nm, PDI 0.18, ZP: −24.46 mV	*L. amazonensis*	Promastigote	62.5 ± 8.3	n.d.	n.d.	[57]
			*L. infantum*		65.9 ± 8.8			
*Copaifera sp.* Linnaeu	Essential oil	Mean size: 76.10 nm, PDI: 0.14, ZP: −2.5 mV	*L. infantum*	Promastigote	16 ± 0.9 (24 h)	Decreased development of lesions, reduction of parasite load in liver and spleen.	Low in vitro toxicity against macrophages	[58]
			*L. amazonensis*		18 ± 0.16 (24 h)			
			*L. infantum*	Amastigote	Diminution of IF (≈90%)			
			*L. amazonensis*		Diminution of IF (≈96%)			
*Carapa guianensis* Aublet	Essential oil	Mean size: 88.17 nm, PDI 0.16, ZP: −3.9 mV	*L. infantum*	Promastigote	366 ± 21 (24 h)	Decreased development of lesions, reduced parasite load in liver and spleen, and improved histopathological features.	Low in vitro toxicity against macrophage	[58]
			*L. amazonensis*		590 ± 23 (24 h)			
			*L. infantum*	Amastigote	Diminution of IF (~50%)			
			*L. amazonensis*		Diminution of IF (~90%)			
*Citrus sinensis*	Essential oil	Mean size of 225 nm (nanoemulsion-based nanogel)	*L. tropica*	Promastigote	151.13	n.d.	n.d.	[59]
			*L. major*		108.31			
*Cinnamomum zeylanicum*	Essential oil	Mean size: 52 nm	*L. tropica*	Promastigote	n.d.	n.d.	n.d.	[60]
			*L. major*					
*Lavandula* *angustifolia*	Not shown	Mean size: 104.2 nm, PDI 0.312, negative ZP: −15.8 mV	*L. major*	Promastigote	0.11	n.d.	Low in vitro toxicity against J774	[61]
				Amastigote	0.06			
*Rosmarinus officinalis*	Not shown	Mean size: 98.7 nm, PDI 0.298, ZP: −17.3 mV	*L. major*	Promastigote	0.08	n.d.	Low in vitro toxicity against J774	[61]
				Amastigote	0.06			

Abbreviatures: polydispersity index (PDI); zeta potential (ZP); infection index (IF); not determined (n.d.); macrophages cell (J774).

**Table 2:** Plant-based liposomal formulations as leishmanicidal agents.

Species	Vegetable Derivative	Physicochemical Characteristics	*Leishmania* spp.	Life Stage	IC50 (µg·mL^−1^)	In Vivo Effect	Toxicity	Reference
*Berberis vulgaris* and *Berberis aristata*	Berberine	Mean size: 120 nm, ZP: −38 mV, and loaded 6 nmol/µmol lipid.	*L. infantum*	Promastigote	6.8	LP enhanced accumulation in liver and spleen; reduced parasitic load (up to 99%)	Sevenfold decrease in toxicity for macrophages	[75]
				Amastigote	1.4			
*Curcuma longa*	Cortex—crude methanol extract	Not shown	*L. amazonensis*	Promastigote	5.5	n.d.	n.d.	[76]
*Curcuma longa*	Curcumin	Mean size: 176.5 nm, PDI: 0.187, mean ZP: +34.99 mV, EE of 92%	*L. major*	Promastigote	2.33	n.d.	HFF cell line treated with nanoliposomes demonstrated biocompatibility	[77]
*Amburana cearensis*	Coumarin derivative (3-(1,3-benzodioxol-5-yl)-2-oxo-2H-chromen-6yl acetate)	Mean size: 173.6 nm, EE 93.2%	*L. major*	Promastigote	Ranged from 10 to 15	Reduced footpad thickness and lower lymph-node parasite load compared to untreated mice	Nanoliposome formulation showed less toxicity to the host cell J774	[33]

Abbreviatures: liposomes (LP); polydispersity index (PDI); zeta potential (ZP); encapsulation efficiency (EE); human primary foreskin fibroblast cells (HFF); macrophage cell (J774); not determined (n.d.).

**Table 4:** Plant-based polymeric nanoparticles as antileishimanicidal agents.

Species	Vegetable Derivative	Physicochemical Characteristics	*Leishmania* spp.	Life Stage	IC50 (µg·mL ^−1^)	In Vivo Effect	Toxicity	Reference
*Apis mellifera*	Red propolis extract	Mean size: 280.2 nm, PDI 0.089, ZP: −26.8 mV and, EE 81.2% flavonoids	*L. braziliensis*	Promastigote	31.2	n.d.	n.d.	[104]
*Aesculus hippocastanum*	β-aescin (preparation of NPs the commercially available was used)	Mean size: 261.4 nm, PDI 0.12, ZP: −24.7 mV and, EE of 32%	*L. infantum*	Amastigote	1.04	n.d.	Lytic effects of β-aescin on the investigated MRC-5 cells were blocked by encapsulating in PLGA NPs (17.87 µg∙mL^1^)	[106]
-	Betulinic acid	Mean size: 112 nm, PDI 0.3, ZP: 8 mV, and EE 93%	*L.* *major*	Amastigote and promastigote	n.d.	Wound healing. Decrease in parasite burden on lesion, liver, and spleen	The highest dose of BA NPs 20 mg/kg: no changes in serum concentrations of BUN, AST, ALT, and ALP; no morphological changes in liver, kidney, and spleen	[107]
*Matricaria chamomilla* L.	Floral chapters—essential oil	Mean size: 801 nm; PDI 0.3, spherical shaped, and EE 89.9%	*L. amazonensis*	Amastigote	14.3	n.d.	Essential oil encapsulation reduces toxic effects on cell lines HaCat, macrophages, and Vero (increase CC_50_)	[108]
				Promastigote	7.2			
*Nigella sativa*	Fixed oil	Mean size: 202–389 nm, PDI 0.08–0.16, ZP of −4.92 (−8.29) mV, and EE ≈ 87%	*L. infantum*	Amastigote	102	n.d.	No cytotoxic effect in vitro	[109]
				Promastigote	159			

Abbreviatures: polydispersity index (PDI); zeta potential (ZP); efficiency encapsulation (EE); blood urea nitrogen (BUN); aspartate transaminase (AST); alanine transaminase (ALT); alkaline phosphatase (ALP); betulinic acid nanoparticles (BA NPs); not determined (n.d.); 50% cytotoxic concentration (CC50).
